# Reliable Reference Genes for Gene Expression Assessment in Tendon-Derived Cells under Inflammatory and Pro-Fibrotic/Healing Stimuli

**DOI:** 10.3390/cells8101188

**Published:** 2019-10-01

**Authors:** Enrico Ragni, Carlotta Perucca Orfei, Annie C. Bowles, Laura de Girolamo, Diego Correa

**Affiliations:** 1 IRCCS Istituto Ortopedico Galeazzi, Laboratorio di Biotecnologie Applicate all’Ortopedia, Via R. Galeazzi 4, I-20161 Milano, Italy; enrico.ragni@grupposandonato.it (E.R.); carlotta.perucca@grupposandonato.it (C.P.O.); 2 Department of Orthopedics, UHealth Sports Medicine Institute, University of Miami Miller School of Medicine, Miami, FL 33136, USA; acb233@miami.edu; 3 Diabetes Research Institute & Cell Transplant Center, University of Miami Miller School of Medicine, Miami, FL 33136, USA; 4 Department of Biomedical Engineering, College of Engineering, University of Miami, Miami, FL 33136, USA

**Keywords:** tendon, tenocyte, progenitor cell, inflammation, reference gene, qRT-PCR

## Abstract

Tendon cells (TCs) are important for homeostatic maintenance in the healthy tendon and to promote tissue healing after injury. Further, resident and rare populations of tendon stem/progenitor cells, located at various sites within the tendon, contribute to tendon recovery by differentiating into repairing TCs. Gene expression analysis, through quantitative reverse-transcription polymerase chain reaction (qRT-PCR), constitutes a useful tool to study cellular responses, including the transition from initial inflammation to healing processes. A critical step required for data normalization is the choice of reliable reference genes (RGs), a process highly underestimated in tendon biology. In this study, the suitability of five commonly used RGs (*ACTB*, *B2M*, *GAPDH*, *HPRT1*, and *RPLP0*) was evaluated using TCs samples cultured in both standard and progenitor-enriching conditions, as well as under either inflammatory (IFNγ + TNFα) or pro-fibrotic/healing (CTGF) stimulation. The stability of the candidate RGs was computationally determined using NormFinder, geNorm, BestKeeper, and DeltaCt applets. Overall, *ACTB* resulted as the most stable RG on the basis of the integration of each gene weight, whereas *B2M* and *RPLP0* performed poorly. To further validate *ACTB*’s optimal performance, we evaluated the expression of *ICAM1*, coding for an immune-related cell surface glycoprotein, and *COL1A1*, encoding collagen type I that is the main component of the tendon extracellular matrix (ECM), both known to be modulated by inflammation. The expression of both genes was heavily affected by the RGs used. Consequently, when analyzing gene expression in tendon-derived cells subjected to various stimulatory protocols, the use of a suitable RG should be considered carefully. On the basis of our results, *ACTB* can be reliably used when analyzing different TC types exposed to pathological conditions.

## 1. Introduction

Tendinopathies can be described as a “continuum” of disturbances historically associated with progressive “wear and tear” of the tendon resulting from aging, overuse, and other causes [1]. As a well-established paradigm, while physiologic loads are required to maintain tendon homeostasis, abnormal loading can lead to tendon injury, through either an acute trauma or a degenerative process resulting from an accumulation of micro-damage and an altered cell/matrix balance [2]. In this frame, pioneering works completed a roadmap of cellular and molecular cascades involved in tendon mechanobiology for both healthy and injured tissues [2]. In addition, more recently, immune/inflammatory responses have also been associated with tendon pathophysiology, complementing the original degenerative descriptions [3]. In fact, tendon injuries, along with mechanical stress, disturb the homeostatic balance between specialized tendon cells (TCs), named tenocytes, and immune cell compartments within the tissue [3]. Resident TCs account for a heterogeneous population of tenocytes, approximately 5% of the total tissue volume, with their main function related to the maintenance of tissue homeostasis [2]. A rare (<1%) subpopulation of the general TCs with stem-like properties has been described as tendon stem/progenitor cells (TSPCs) [4], although a large debate is still ongoing regarding a proper definition and characterization of this subset. Various strategies have helped elucidate their identification, including cell surface marker selection (e.g., CD146 positivity) [5] and culture conditions (e.g., hypoxia and PGE_2_ supplementation) [6]. Furthermore, the injection of connective tissue growth factor (CTGF) has been reported to enrich this population during early phases of tendon healing and promote tissue remodeling [5,7,8].

For both general TCs and specific TSPCs, a continuously expanding body of literature is starting to shed light on cellular and molecular mechanisms, aiming at deciphering their roles during acute/chronic injuries and healing responses [9,10]. Gene expression analysis, specifically qRT-PCR, has been used for decades as an informative tool to dissect such mechanisms, as it provides information regarding the different cellular responses to stimulatory environments. To consistently evaluate genes relative expression, the choice of reliable reference genes (RGs) is mandatory, as inappropriate RGs can lead to erroneous data and misleading experimental results [11]. An ideal RG must be stably expressed in all test samples, exhibiting low variability among them [12]. Unfortunately, it has become clear that the most common housekeeping genes used for many years for normalization do not meet those criteria, showing variations in the given conditions [13,14]. Therefore, it is necessary to carefully identify stable RGs to be tested and validated with different sets of cells and in multiple conditions.

To date, despite several RGs being considered “gold standards” and used in the analysis of tendons and derived cells, there is no consensus on reliable candidates. In a recent work, the suitability of six reference genes (*18S*, *ACTB*, *B2M*, *GAPDH*, *HPRT1*, and *TBP*) using samples from rotator cuff tendons was assessed, with *HPRT1* resulting the most reliable [15]. In normal and diseased horse tendons, 12 commonly used RGs were analyzed, *GAPDH* being the most stable followed by *ACTB* [16]. Regarding human TCs treated with tenogenic supplements, *YWHAZ* and *RPL13A* showed superior consistency [17]. Even though these reports provide valuable information, their intrinsic distinct nature (i.e., different organisms, tissues and isolated cells, and the presence or absence of exogenous supplements) limits their use to describe a “universal” RG to study tendon cell biology, especially when dealing with its various cellular components.

For this reason, the aim of this work was to identify stable RGs in human tendon-derived cells cultured at both high and low densities, reminiscent of the described general TCs [18] and of enriched TSPCs, respectively, as the latter culturing condition has demonstrated to increase the expression of the progenitor marker *OCT4* [19] Furthermore, in order to in vitro model various aspects of tendinopathy, those cells were exposed to either inflammatory (IFNγ + TNFα) or pro-fibrotic/healing (CTGF) stimulation. To obtain reliable candidates for the different cell types and distinct culture conditions, four computational gene expression analysis packages were used for the first time on tendon cells (geNorm, NormFinder, BestKeeper, and DeltaCt). The results obtained with this systematic approach will become a useful technical tool for future studies aimed at dissecting the molecular underpinnings of tendon biology and healing by reliably assessing gene expression.

## 2. Materials and Methods

### 2.1. Tendon Dissection and Cell Isolation

Human tendon cells were isolated from discarded fragments of the semitendinosus and gracilis tendons harvested from three de-identified patients (*n* = 3, males, 33 ± 9 years old) who underwent elective anterior cruciate ligament (ACL) reconstruction using hamstring tendons and provided their written informed consent (M-SPER-015- Ver. 2 - 04.11.2016 for the use of surgical waste material). The protocol was reviewed and approved by IRCCS Istituto Ortopedico Galeazzi IRB. After 16 h of enzymatic digestion with 0.3% *w*/*v* type I collagenase (185 U/mg, Worthington Biochemical Corporation, Lakewood, NJ, USA) [20], the samples were filtered through a 100 μm cell strainer (Becton, Dickinson and Co., NJ, USA) and centrifuged (300× *g*, 5 min). The resulting cells were plated at a density of either 5000 cells/cm^2^ (high density, HD) or 50 cells/cm^2^ (low density, LD) in low-glucose DMEM supplemented with 10% and 20% FBS, respectively, and maintained in an incubator at 37 °C in a humidified atmosphere with 5% CO_2_. Cells at passage 2 were treated with either IFNγ + TNFα (10 ng/mL and 15 ng/mL) or CTGF (10 ng/mL) for 48 h before further processing.

### 2.2. Immunofluorescence/Immunohistochemistry Analysis

Cells were fixed with 10% neutral buffered formalin, washed with PBS, and then treated with a blocking solution containing 2% bovine serum albumin (BSA, Sigma-Aldrich, St. Louis, MA, USA). A primary antibody for CD146 (ab134065, Abcam, Cambridge, MA, USA) was diluted in 1% BSA (1:500) and incubated overnight at 4 °C with the cells. The samples were then washed three times with 1% Tris-buffered saline (Sigma-Aldrich) and incubated with the secondary antibody Goat anti-Rabbit IgG (H + L) highly cross-adsorbed, (Alexa Fluor Plus 647, 1:1000) for 1 h at room temperature. The cells were then washed and incubated with 0.1 µg/mL DAPI (DNA stain, Abcam) for 5 min.

The negative controls were subjected to overnight incubation at 4 °C with blocking buffer without the primary antibody. Microscope images were acquired using a Leica DMi8 microscope with Leica LAS X software (Leica, Heidelberg, Germany).

### 2.3. RNA Extraction and qRT-PCR Validation Studies

RNA was extracted using RNeasy Mini Kit (Qiagen, Frederick, MD, USA). RNA purity was assessed by 260/280 nm and 260/230 nm absorbance ratios and resulted to be 2.16 ± 0.01 and 1.98 ± 0.13, respectively. cDNA synthesis was performed using SuperScript™ VILO™ cDNA synthesis kit (Invitrogen, Carlsbad, CA, USA). qRT-PCR was performed using RT^2^ SYBR Green qPCR Mastermix (Qiagen) reagent and transcript-specific primers (RT^2^ qPCR primer assay for human *ACTB* (Product # PPH00073G), *B2M* (PPH01094E), *COL1A1* (PPH01299F), *GAPDH* (PPH00150F), *HPRT1* (PPH01018C), *ICAM1* (PPH00640F), and *RPLP0* (PPH21138F), Qiagen), following the manufacturer’s instructions. For each sample, independent qRT-PCR were performed using a StepOnePlus real-time PCR system thermocycler (Applied Biosystems, Thermo Fisher Scientific, Waltham, MA, USA). Amplification was obtained using the following cycling conditions: 10 min at 95 °C, followed by 40 cycles of 15 s at 95 °C and 1 min at 60 °C. As a quality control, we generated a first derivative dissociation curve for each well under the following conditions: 95 °C, 1 min; 65 °C, 2 min; 65 °C to 95 °C increasing by 2 °C/min. No more than one peak appeared in each reaction well, confirming amplification specificity. *ACTB*, *B2M*, *GAPDH*, *HPRT1*, and *RPLP0* were analyzed as reference genes, and the most stable (*ACTB*) and the most variable (*B2M* and *RPLP0*) genes were used to score *COL1A1* and *ICAM1* modulation across samples with the ΔΔCt (cycle threshold) method.

### 2.4. Data Analysis

RGs expression stability was estimated using four computational gene expression analysis packages: NormFinder, geNorm, BestKeeper, and DeltaCt. The raw Ct values were used directly for stability calculations in BestKeeper analysis and DeltaCt method and converted into relative quantities before being imported into the geNorm and Norm-Finder applets. geNorm scores the average pairwise variation of an RG versus all other genes in the given samples [13]; NormFinder calculates the expression stability value based on inter- and intra-group variation [21]; the stability ranking of a candidate reference gene is determined by the CV (coefficient of variation) and SD (standard deviation) values in BestKeeper [22]; the DeltaCt method compares the relative expression of “pairs of genes” within each sample to confidently identify useful RGs [23]. Each algorithm generates the ranking of the RG according to their stability, assigning a series of continuous integers starting from 1. To improve the outcome, the geometric mean (geomean) of each gene weight generated by the four approaches was further calculated. The RG with the lowest geomean was considered to be the most stable.

Hierarchical clustering and principal component analysis (PCA) were obtained with the ClustVis webtool after row centering under the following settings for both rows’ and columns’ clustering distance and method: correlation and average, respectively [24].

### 2.5. Statistical Analysis

Statistical analysis was performed using GraphPad Prism v5.0 software (GraphPad Software Inc., La Jolla, CA, USA). *COL1A1* and *ICAM1* normal distribution of values was assayed by Kolmogorov–Smirnov normality test prior to a Student’s *t*-test. Differences were considered significant at *p*-value < 0.05.

## 3. Results

### 3.1. Tendon Cells Isolation and Characterization

**Figure 1:**
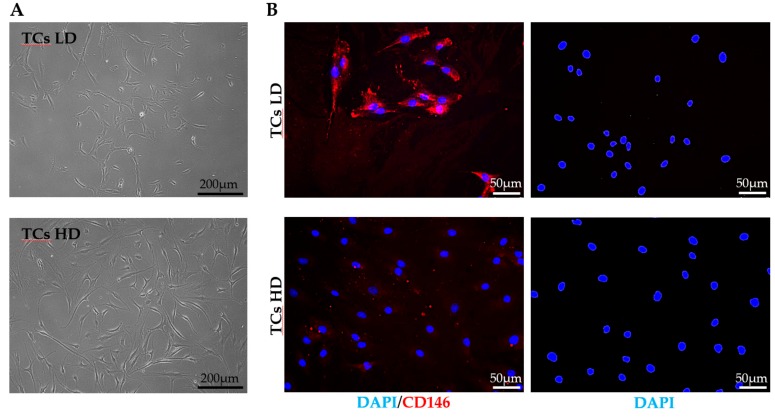
Tendon cells’ (TCs) morphology and CD146 expression. (**A**). Representative bright-field pictures of low-density (LD) and high-density (HD) TCs. (**B**). Immunofluorescence staining of LD and HD TCs for CD146 (red) and DAPI (blue). Negative control samples were subjected to overnight incubation at 4 °C with blocking buffer without the primary antibody.

Tendon-derived primary cells seeded at 5 × 10^1^ cells/cm^2^ (LD) showed isolated colonies with small cobblestone-like cells, while at 5 × 10^3^ cells/cm^2^ (HD), a homogeneous growth surface with fusiform and fibroblast-like morphology was visible (Figure 1A). These distinct cellular morphologies were complemented phenotypically with a clear difference in the expression of the surface antigen marker CD146. As expected, and on the basis of TSPCs enrichment in low-density conditions, LD cells exhibited a marked positivity for CD146 (87% ± 6 positive). Contrarily, HD conditions, reminiscent of heterogeneous TCs, allowed only a very low and rare CD146 expression (8% ± 3) (Figure 1B). These results reinforced the idea of selecting by culture at different cell densities two tendon-derived cell populations, supporting the subsequent gene expression examination.

### 3.2. Expression of Candidate RGs

**Figure 2:**
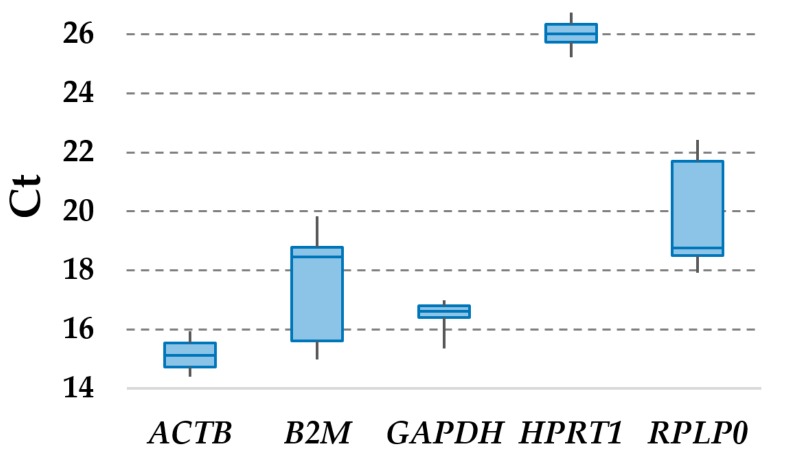
Ct values of tested reference genes (RGs) across tendon cell samples.

Considering all TC samples, with and without IFNγ + TNFα or CTGF treatment, *ACTB* had the highest expression (lowest Ct values), whereas *HPRT1* had the lowest (Figure 2 and Table 1). Setting a Ct cut-off of 25 for weak expression, with the only exception of *HPRT1*, all candidates RGs were detected as highly to moderately expressed. Moreover, to reduce the likelihood of including co-regulated RGs in the study, we performed a contiguity analysis, which evidenced that none of them resides within the same gene cluster. *ACTB* is located on chromosome (chr) 7, *B2M* on chr15, *HPRT1* on chrX, and *GAPDH* and *RPLP0* on chr12, spatially separated at the two extremities of the chromosome (position 6,534,517–6,538,371 for *GAPDH* and 120,196,699–120,201,211 for *RPLP0*; total chr12 length is 133,275,309 bp, with 998 genes, centrosome position 35.5 Mbp) and, therefore, unlikely to reside in the same cluster, usually encompassing less than 10 genes or, in rare cases, up to 100 [25].

### 3.3. Stability Analysis of RGs

The stability rankings of the selected RGs in LD and HD TCs, under both resting (naive) conditions and IFNγ + TNFα or CTGF exposure is shown in Table 2. Each category represents the whole dataset under the indicated treatment or seeding density, namely, 27 samples for “ALL” and 9 samples for all the other groups. Since the used approaches generated few differences in outcomes, integration and normalization of the data were applied, generating the comprehensive geomean value. In LD, the best RG resulted to be *ACTB* (geomean of 1.32), and the least stable one was *B2M* (4.73), whereas considering HD cells, *GAPDH* performed very well (1), whereas *RPLP0* showed great variability (4.73). Further, focusing on the culturing condition, in naïve (non-primed) tendon cells, *GAPDH* (1) was the most stable, and *B2M* (4.73) was the least stable. Under inflammatory conditions (IFNγ + TNFα), *HPRT1* performed good (1.32), and *B2M* again scored last (3.98). When CTGF was supplied, *RPLP0* (1.19) behaved as a reliable RG, and *HPRT1* was the least reliable (5). Eventually, since both seeding densities and culturing conditions gave rise to slightly different outcomes, all samples were scored together. In this analysis, *ACTB* (1.32) resulted the best performer, and *RPLP0* (4.73) the less reliable RG.

**Figure 3:**
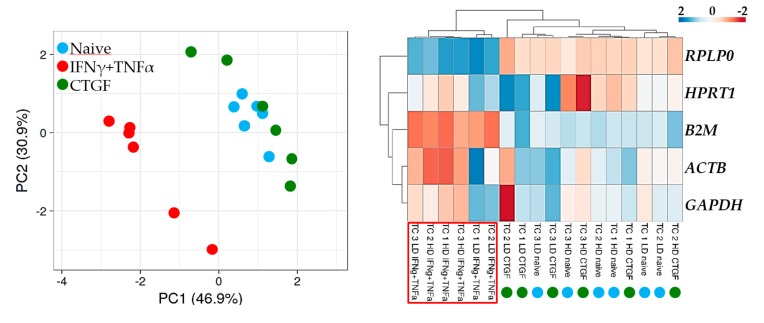
Principal component analysis (PCA) and heat map of RG expression values. In PCA, the *X*- and *Y*-axis show principal component 1 and principal component 2 that explain 46.9% and 30.9% of the total variance, respectively. In the heatmap, positive values mean higher Cts, and negative values mean lower Cts with respect to mean values after row centering for each RG, indicating lower (high CTs) or higher (low Cts) basal expression, respectively. Both rows and columns were clustered using correlation distance and average linkage.

Then, the samples were analyzed by PCA and hierarchical clustering (HC) to score major differences between populations (Figure 3). PCA showed that the expression pattern of the IFNγ + TNFα samples significantly differed, with all inflammation samples grouped apart. Further, HC confirmed the PCA data and emphasized how this outcome may be associated with the consistently higher *B2M* (low Ct values) and lower *RPLP0* (high Ct values) levels under inflammation, as observed in Figure 3. Notably, with both approaches, CTGF supplementation was not able to influence TCs signature, which indicated the absence of major effects on transcriptional regulation of the selected RGs.

### 3.4. Impact of RGs Choice on the Expression Levels of Target Genes

qRT-PCR assays on *ICAM1* and *COL1A1* were performed in order to further evaluate the reliability of the selected candidate RGs in the TCs samples (LD and HD). *ICAM1* and *COL1A1* expression levels were normalized using the most stable (*ACTB*) and the two least stable (*B2M* and *RPLP0*) RGs. First, we evaluated the modulation of selected genes between the different seeding densities using LD values as a reference. On the basis of *ACTB* expression, we found statistical significant change (*p*-value = 0.0128 with ratio of 0.30) for *ICAM1* in HD samples. For this condition, also the less reliable RGs showed statistically significant modulation (fold < 0.5, *p*-value < 0.05). When comparing the fold change given by the different RGs, no significant (ratio lower than 0.5 or higher than 2, *p*-value < 0.05) changes were found, meaning that with respect to *ICAM1* expression in the absence of external stimuli, all three RGs behave with a similar trend. Regarding *COL1A1*, no differences (fold > 2, *p*-value < 0.05) were scored between both samples and the different RGs.

**Figure 4:**
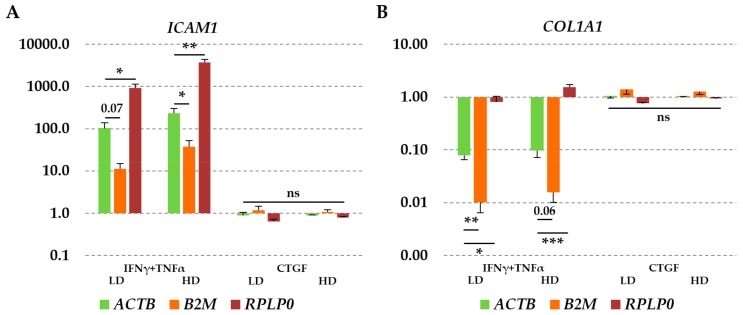
*ICAM1* (**A**) and *COL1A1* (**B**) modulation under IFN*γ* + TNFα or connective tissue growth factor (CTGF) supplementation is dependent on the selected RGs. Increase in *ICAM1* and decrease in *COL1A1* expression with respect to untreated cells are significantly different depending on which RG (*B2M* or *RPLP0*) is used. Values are expressed as fold-change mean ± SEM, *n* = 3 independent cell isolates; ns: non-significant, * for *p*-value < 0.05, ** for *p*-value < 0.01, *** for *p*-value < 0.001.

When cytokine supplementation was assessed, the choice of the RGs became crucial for IFNγ + TNFα samples, whereas again for CTGF, no differences were scored. Under inflammatory conditions, on the basis of *ACTB* expression, a marked increase of *ICAM1* and a decrease of *COL1A1* were observed in all seeding conditions (Figure 4A, Table 3). The gain of *ICAM1* was maintained also when considering the least stable RG, although the use of *B2M* resulted in an underestimation and that of *RPLP0* led to an overestimation of mRNA abundance, always significantly different from (ratio lower than 0.5 or higher than 2, *p*-value < 0.05 or < 0.07 for *B2M* with respect to *ACTB* in LD) and therefore erroneous for *ACTB* values. For *COL1A1* (Figure 4B, Table 3), the effect of unreliable RGs was the opposite, with apparently higher downregulation observed in inflammatory conditions using *B2M* and almost an absence of modulation using *RPLP0*, being all these data again significantly different with respect to *ACTB* (with the only exception of *B2M* with respect to *ACTB* in HD cells, *p*-value of 0.06). Therefore, when IFNγ + TNFα were added to the culture medium, the effect of RGs choice gets decisive for the correct evaluation of target mRNA abundance, in term of both modulation and amount of variation.

## 4. Discussion

Herein, a rigorous method to determine and validate RGs in tendon cells identified *ACTB* as the most stable gene, whereas *B2M* and *RPLP0* performed poorly. Incorrect RGs choice affected the reliable evaluation of the expression of the target genes *ICAM1* and *COL1A1*.

In this work, five candidate genes were selected for identifying the best reference among 18 TCs samples, grown in both standard (HD) and progenitor-promoting (LD) density seeding, under different experimental conditions, mimicking both inflammation (IFNγ + TNFα) and pro-fibrotic/healing phases (CTGF) in vitro. Four computational gene expression analysis packages (geNorm, NormFinder, BestKeeper and DeltaCt) were used to reliably determine the best candidate(s) for all conditions. The selection of the packages was based on their alternative approaches: i) the average pairwise variation of a reference gene versus all other genes among the given samples is scored by geNorm [13]; ii) NormFinder calculates the expression stability value based on intra- and inter-group variation [21]; iii) in BestKeeper, the stability ranking of a candidate reference gene is determined by the CV and SD values [22]; iv) the DeltaCt method relies on the ΔCt approach, which requires less use of specialist programs by comparing pairs of candidate genes [23]. Since none of these tools is considered to be superior in terms of outcome power, the geometric mean method of the ranking values was used to obtain a reliable consensus.

A fundamental requisite to be an optimal RG is a stable expression regardless of changes in the environment, physiological conditions, and cell type, especially those under study. Consequently, an RG with known or suspected participation in biological phenomena that is part of the study should be properly validated a priori. A typical example is *GAPDH*. A pivotal study encompassing a panel of 72 normal human tissues from 618 donors showed no differences in gene expression between males and females or with donor age [26]; however, variations were observed in tissues when associated with the role of *GAPDH* within the glycolytic pathway. In this frame, the five RGs chosen for our analysis have crucial physiological roles in essential pathways, supporting their choice for an a priori superior stability under different treatments. *ACTB* encodes beta-actin, which is essential for a number of cytoplasmic functions; *B2M* codes for the MHC class I cell surface molecule beta-2-microglobulin, a cell surface marker for all nucleated cells; *GAPDH* is one of 10 enzymes that catalyze reactions in the glycolytic pathway; *HPRT1* plays a central role in the generation of purine nucleotides and *RPLP0* encodes the 60S acidic ribosomal protein P0, a component of the 60S ribosomal subunit.

Under these premises, in our experimental settings, it clearly emerged that *B2M* and *RPLP0* performed poorly in almost all samples under analysis, both TCs and TSPC-enriched. Notably, *B2M* resulted to behave poorly also in keratinocytes treated with IFNγ [27], suggesting its susceptibility to this molecule, as herein evidenced by hierarchical clustering and PCA. On the contrary, *ACTB*, followed by *GAPDH*, resulted as the most stable RGs. *HPRT1* also showed good performance. These results are consistent with those obtained in tendons where *HPRT1*, *ACTB*, and *GAPDH* were suggested as optimal candidates out of a similar panel of candidates, therefore herein demonstrating for the first time that RGs in tendon cells have a similar performance both in vitro and in vivo [15,16]. In this frame, for isolated tenocytes, the only published report encompassed a different selection of RGs with respect to our study, with only *GAPDH* that was shared but did not perform optimally [17]. Due to the great difference in the RGs scored and the presence of exogenous tenogenic cytokines, it is difficult to make a direct comparison and assess whether the variance is due to the factors used or to a dissimilarity in the analytical methods.

To validate the suitability of *ACTB* identified in this study, the relative expression levels of *ICAM1* and *COL1A1* under the different experimental conditions were assessed. In tenocytes, *ICAM1* was previously reported to significantly increase upon stimulation with IFNγ + TNFα [9]. Moreover, the elevation of TNFα with a concomitant downregulation of *COL1A1* has been found within stressed tendons [28] and, further, TNFα-inhibitory responsive element (TαRE) has been mapped in the upstream *COL1A1* promoter region [29]. Thus, these two genes were perfect candidates to score selected RGs performance. Consistently, under IFNγ + TNFα, using *ACTB* as a RG, we were able to confirm *ICAM1* increase and *COL1A1* downregulation in both TCs and TSPC-enriched cultures. The results revealed that their expression pattern was significantly over- or underestimated when using the unstable genes for normalization (*B2M*/*RPLP0*), or even completely abolished when *COL1A1* was normalized with respect to *RPLP0*, suggesting that the selection of appropriate, stable RGs is crucial for the correct normalization of qRT-PCR data in inflamed tenocytes. On the contrary, when CTGF was supplemented, no major RGs modulation, at least for the assayed candidates, was observed. Notably, in our experimental setting, CTGF was not able to induce significant *COL1A1* upregulation as previously reported [5]. This may be due to both the difference in cytokine concentration, 10 ng/mL vs. 100 ng/mL, and the stimulation time, 2 days vs. 14 days. This suggests a concentration- and time-dependent relationship for CTGF-dependent *COL1A1* increase. Further studies will be crucial to shed light on this molecular mechanism that, to date, has been poorly deciphered in tendon cells.

## 5. Conclusions

To the best of our knowledge, this study constitutes the first systematic evaluation of a set of candidate RGs as normalization factors in qRT-PCR analysis for tendon-derived cells, also under inflammatory- and pro-fibrotic/healing-like environments in vitro. The results of a comprehensive ranking order showed that *ACTB* displayed the highest stability across the set of samples in all conditions. The expression analysis of *ICAM1* and *COL1A1* emphasized the importance of suitable RGs to get accurate and reliable quantitation by qRT-PCR. These results are of great importance for further gene expression analyses in tendon-derived cells, facilitating the dissection of underlying molecular mechanisms related to tendon stress and healing responses. In fact, several molecular cascades have been postulated as critical, controlling local modulation by both TCs and TSPCs, and thus require detailed descriptions. In this frame, gene expression still constitutes an important assessment tool when dissecting molecular mechanisms. Therefore, the selection of reliable RGs for normalization in human tendon-derived cells warrants analytical accuracy, and the herein presented results are of crucial importance for future studies.

## Figures and Tables

**Table 1:** Ct values of tested RGs across tendon cell samples.

	*ACTB*	*B2M*	*GAPDH*	*HPRT1*	*RPLP0*
***TC 1 LD naive***	15.04	18.68	16.78	26.07	19.47
***TC 2 LD naive***	15.12	18.41	17.05	26.06	19.32
***TC 3 LD naive***	15.58	18.16	17.19	26.20	19.22
***TC 1 LD IFNγ + TNFα***	15.94	15.55	17.34	26.53	22.96
***TC 2 LD IFNγ + TNFα***	15.12	15.08	17.32	26.39	22.47
***TC 3 LD IFNγ + TNFα***	14.68	15.10	16.66	26.12	22.27
***TC 1 LD CTGF***	15.58	19.82	17.19	26.61	19.28
***TC 2 LD CTGF***	14.64	18.20	15.73	26.73	18.45
***TC 3 LD CTGF***	15.71	18.48	17.36	26.73	19.05
***TC 1 HD naive***	15.46	18.74	16.99	25.64	19.08
***TC 2 HD naive***	15.26	18.99	16.99	25.74	18.98
***TC 3 HD naive***	15.26	18.94	16.82	25.47	19.67
***TC 1 HD IFNγ + TNFα***	14.40	14.98	16.59	25.70	22.65
***TC 2 HD IFNγ + TNFα***	14.41	15.26	16.80	25.88	22.12
***TC 3 HD IFNγ + TNFα***	14.61	15.80	16.54	25.91	22.62
***TC 1 HD CTGF***	15.61	18.75	17.15	25.74	19.03
***TC 2 HD CTGF***	15.08	19.48	17.05	25.97	18.79
***TC 3 HD CTGF***	14.86	18.80	16.76	25.21	18.97

**Table 2:** Stability rankings of tested RGs.

	Geomean		DeltaCt		BestKeeper		NormFinder		geNorm	
***ALL***	*ACTB*	1.32	*ACTB*	1.08	*GAPDH*	0.29	*ACTB*	0.159	*ACTB GAPDH*	0.318
	*GAPDH*	1.41	*GAPDH*	1.08	*HPRT1*	0.34	*GAPDH*	0.159		
	*HPRT1*	2.71	*HPRT1*	1.16	*ACTB*	0.37	*HPRT1*	0.265	*HPRT1*	0.468
	*B2M*	4.23	*B2M*	2.14	*RPLP0*	1.51	*B2M*	2.062	*B2M*	1.097
	*RPLP0*	4.73	*RPLP0*	2.17	*B2M*	1.55	*RPLP0*	2.099	*RPLP0*	1.527
***NAIVE***	*GAPDH*	*1*	*GAPDH*	0.3	*GAPDH*	0.11	*GAPDH*	0.095	*ACTB GAPDH*	0.149
	*ACTB*	1.68	*ACTB*	0.33	*ACTB*	0.16	*ACTB*	0.185		
	*RPLP0*	3.22	*RPLP0*	0.39	*RPLP0*	0.2	*RPLP0*	0.272	*HPRT1*	0.254
	*HPRT1*	3.94	*HPRT1*	0.4	*B2M*	0.25	*HPRT1*	0.322	*RPLP0*	0.32
	*B2M*	4.73	*B2M*	0.46	*HPRT1*	0.25	*B2M*	0.404	*B2M*	0.377
***IFNγ + TNFα***	*HPRT1*	1.32	*HPRT1*	0.31	*RPLP0*	0.23	*HPRT1*	0.099	*GAPDH HPRT1*	0.164
	*GAPDH*	2	*GAPDH*	0.34	*B2M*	0.25	*GAPDH*	0.213		
	*RPLP0*	2.45	*RPLP0*	0.37	*HPRT1*	0.26	*RPLP0*	0.244	*ACTB*	0.277
	*ACTB*	3.94	*ACTB*	0.42	*GAPDH*	0.3	*ACTB*	0.348	*RPLP0*	0.333
	*B2M*	3.98	*B2M*	0.45	*ACTB*	0.45	*B2M*	0.393	*B2M*	0.381
***CTGF***	*RPLP0*	1.19	*RPLP0*	0.47	*RPLP0*	0.21	*ACTB*	0.128	*ACTB RPLP0*	0.278
	*ACTB*	1.41	*ACTB*	0.48	*ACTB*	0.39	*RPLP0*	0.139		
	*GAPDH*	3	*GAPDH*	0.55	*GAPDH*	0.42	*GAPDH*	0.385	*GAPDH*	0.324
	*B2M*	4	*B2M*	0.66	*B2M*	0.49	*B2M*	0.53	*B2M*	0.45
	*HPRT1*	5	*HPRT1*	0.81	*HPRT1*	0.53	*HPRT1*	0.748	*HPRT1*	0.594
***LD***	*ACTB*	1.32	*ACTB*	1.08	*HPRT1*	0.24	*ACTB*	0.164	*ACTB GAPDH*	0.328
	*GAPDH*	1.68	*GAPDH*	1.12	*GAPDH*	0.38	*GAPDH*	0.164		
	*HPRT1*	2.28	*HPRT1*	1.16	*ACTB*	0.39	*HPRT1*	0.227	*HPRT1*	0.466
	*RPLP0*	4.23	*RPLP0*	2.17	*B2M*	1.5	*RPLP0*	2.091	*RPLP0*	1.104
	*B2M*	4.73	*B2M*	2.21	*RPLP0*	1.53	*B2M*	2.136	*B2M*	1.545
***HD***	*GAPDH*	1	*GAPDH*	1.04	*GAPDH*	0.17	*GAPDH*	0.149	*GAPDH HPRT1*	0.298
	*HPRT1*	1.68	*HPRT1*	1.1	*HPRT1*	0.17	*HPRT1*	0.149		
	*ACTB*	3	*ACTB*	1.1	*ACTB*	0.38	*ACTB*	0.151	*ACTB*	0.381
	*B2M*	4.23	*B2M*	2.14	*RPLP0*	1.5	*B2M*	2.08	*B2M*	1.034
	*RPLP0*	4.73	*RPLP0*	2.29	*B2M*	1.6	*RPLP0*	2.237	*RPLP0*	1.535

For BestKeeper and DeltaCt SD, for NormFinder SV, and for geNorm M values are shown; geomean value is the geometric mean. The lower the value, the higher the stability. NB: geNorm applets released a couple of best RGs.

**Table 3:** *ICAM1* and *COL1A1* modulation in single samples and their general patterns.

		*ACTB*		*B2M*		*RPLP0*	
		*ICAM1*	*COL1A1*	*ICAM1*	*COL1A1*	*ICAM1*	*COL1A1*
TC 1 LD	IFNγ + TNFα	175.243	0.058	10.770	0.004	1061.283	0.353
TC 2 LD		53.996	0.105	5.438	0.011	480.558	0.929
TC 3 LD		78.577	0.072	17.617	0.016	1219.303	1.121
***TC LD***		***102.605***	***0.079***	***11.275***	***0.010***	***920.382***	***0.801***
TC 1 HD		175.755	0.065	27.045	0.010	4347.781	1.602
TC 2 HD		154.498	0.077	20.960	0.010	2445.748	1.221
TC 3 HD		368.332	0.149	65.354	0.027	4462.196	1.805
***TC HD***		***232.862***	***0.097***	***37.786***	***0.016***	***3751.908***	***1.543***
TC 1 LD	CTGF	1.117	1.248	1.697	1.908	0.674	0.752
TC 2 LD		0.631	0.912	0.768	1.103	0.481	0.691
TC 3 LD		0.936	1.008	1.077	1.159	0.764	0.822
***TC LD***		***0.895***	***1.056***	***1.181***	***1.390***	***0.640***	***0.755***
TC 1 HD		0.948	1.122	0.859	1.016	0.827	0.978
TC 2 HD		0.824	0.985	1.307	1.562	0.816	0.975
TC 3 HD		0.866	1.032	1.041	1.250	0.704	0.839
***TC HD***		***0.880***	***1.047***	***1.069***	***1.276***	***0.782***	***0.930***

TC 1/2/3 LD or HD stand for the single cell isolates, whereas *****TC LD***** or *****HD***** stands for the general average, as per Figure 4A,B.
